# A SHAP-interpretable XGBoost model: MRI-based intratumoral perfusion heterogeneity predicts HER2-zero, -low, and -positive ternary expression status in breast cancer

**DOI:** 10.1186/s40644-026-01000-4

**Published:** 2026-02-02

**Authors:** Shuxing Wang, Xiaowen Liu, Yudie Pan, Cici Zhang, Yu Wu, Changsi Jiang, Xue Tang, Yan Luo, Jingshan Gong

**Affiliations:** 1 Department of Radiology, Shenzhen People’s Hospital (The First Affiliated Hospital, Southern University of Science and Technology, The Second Clinical Medical College, Jinan University), Shenzhen, 518020 China; 2 Department of Radiology, Shenzhen People’s Hospital, The Second Clinical Medical College, Jinan University, Shenzhen, 518020 China; 3 Department of Radiology, Guangzhou Red Cross Hospital (Guangzhou Red Cross Hospital of Jinan University), Guangzhou, Guangdong China; 4 Guangzhou Women and Children’s Medical Center, Guangdong Provincial Clinical Research Center for Child Health, Guangzhou, China

**Keywords:** Breast cancer, Intratumoral heterogeneity, Radiomics, Human epidermal growth factor receptor 2

## Abstract

**Objectives:**

This study aimed to predict HER2 status (HER2-zero, -low, and -positive) in breast cancer using MRI perfusion heterogeneity. The SHapley Additive exPlanations (SHAP) method was employed to interpret the outputs of machine learning models, which is crucial for guiding treatment with novel antibody-drug conjugates (ADCs).

**Materials and methods:**

The retrospective study included 912 women from three centers (Center A [*n* = 570] as the training cohort, and Centers B [*n* = 173] and C [*n* = 169] as external test cohorts) who underwent MRI between April 2018 and March 2024. Voxel vectors from MRI perfusion parameters (wash-in, wash-out, wash-out ratio) were clustered into subregions using k-means clustering. Radiomics features were extracted, and an XGBoost model incorporating these features was used to build the Habitat model. SHAP was applied to evaluate feature contributions and their importance.

**Results:**

Four sub-regions of tumor perfusion patterns were identified, containing 8, 8, 8, and 10 radiomics features, respectively. The Habitat model achieved AUCs of 0.902 for HER2-zero, 0.877 for HER2-low, and 0.880 for HER2-positive in Center A. In the external test cohorts, AUCs were 0.873, 0.845, and 0.865 for Center B and 0.865, 0.844, and 0.878 for Center C, respectively. SHAP analysis revealed the radiomic features that most strongly contributed to distinguishing HER2-zero, -low, and -positive tumors across the four perfusion-derived subregions. The global SHAP results identified subregion-specific features with the highest influence on model decisions, while the local SHAP explanations clarified how individual feature patterns drove prediction outcomes for specific patients.

**Conclusion:**

The Habitat model accurately predicts HER2-zero, HER2-low, and HER2-positive expression status, while SHAP clarifies the contribution of subregion-derived radiomic features and enhances the overall interpretability and clinical transparency of the prediction framework.

**Clinical trial number:**

Not applicable.

**Supplementary Information:**

The online version contains supplementary material available at 10.1186/s40644-026-01000-4.

## Introduction

Breast cancer is a prevalent malignant tumor among women worldwide, accounting for 11.6% of new cases annually [1]. Human epidermal growth factor receptor 2 (HER-2) is a significant factor influencing the prognosis of patients with breast cancer. It is a crucial gene target for breast cancer treatment [2, 3]. Anti-HER2 treatment for HER2-positive breast cancer has altered the disease’s natural biology. Only 15–20% of breast cancers are considered HER2-positive, up to 55% are considered HER2-low, and 25% as HER2-zero. Immunohistochemistry (IHC) staining and fluorescence in situ hybridization (FISH) determine HER2 expression status in breast cancer [4–6]. Recently, a novel antibody-drug conjugate (ADC) targeting HER2, Trastuzumab Deruxtecan, showed significantly longer progression-free survival and overall survival than conventional chemotherapy in patients with HER2-low metastatic breast cancer [7]. The HER2-low expression population in breast cancer possesses unique biological characteristics, including pathological histology, molecular biological features, neoadjuvant chemotherapy, and treatment prognosis [8–11]. Therefore, traditional binary classification method for distinguishing HER2 status is no longer sufficient; a ternary classification method of subdividing HER2 expression status holds significance.

Owing to excellent soft tissue and spatial resolution, magnetic resonance imaging (MRI) has become an important imaging modality for diagnosing, staging, and treating response assessment of breast cancer [12, 13]. By extracting high-dimensional radiomics features using computers, MRI-based radiomics is a potential imaging biomarker for the intratumoral heterogeneity of breast cancers [14]. Some radiomic features are associated with HER2 expression status in breast cancer [15]. Despite the powerful predictive capabilities of machine learning models due to their complex hyperparameters, their development is still constrained by the “black-box” problem [16, 17]. To address this issue, we use SHapley Additive exPlanation (SHAP) methods to explain ML models, allowing us to visualize the contribution of each feature to the model’s predictions [18]. These methods help clarify individual predictions and analyze the model’s overall behavior, thereby increasing the transparency of machine learning models and facilitating the adoption and acceptance of artificial intelligence technologies in clinical practice.

Although texture features measure tumor heterogeneity to some extent, it is incomplete because its calculation is based on the entire tumor, ignoring regional phenotypic variations within the tumor [19, 20]. Therefore, based on the analysis of the entire tumor, HER2 expression status no longer aligns with the current diagnostic concept. Expression status of HER2 is related to uncontrolled high proliferation [21], requiring strong blood supply. Dynamic contrast-enhanced (DCE)-MRI provides information on tumor vascular perfusion, reflecting the tumor’s highly proliferative state, revealing HER2’s different expression states. Perfusion imaging heterogeneity can resolve tumor subregions in the spatial dimension and is called habitat imaging [19, 22, 23]. Conventional radiomics often relies on whole-tumor features and overlooks spatial variations in perfusion phenotypes that may reflect underlying HER2 biology. To address this limitation, we propose a habitat-based radiomics approach that incorporates voxel-level perfusion heterogeneity into a ternary HER2 classification model. By integrating SHAP analysis, our framework not only improves predictive performance but also provides transparent, clinically interpretable explanations of how perfusion-derived features drive model decisions. Unlike previous studies that focused on binary classification tasks for predicting HER2 expression status or compared the three expression states in pairs [24, 25]. This requires using three task models to obtain the results of HER2 expression states, and the model’s predictive accuracy gradually decreases.

Therefore, we established a ternary classification task to directly evaluate HER2-zero, HER2-low, and HER2-positive expression states while maintaining strong performance in categorization. Simultaneously, we integrated the model with SHAP methods to explain and visualize the classification and prediction processes.

## Materials and methods

### Patient cohorts

This retrospective multicenter study was approved by the institutional review committee of each participating center; the requirement for written informed consent was waived.

**Fig. 1:**
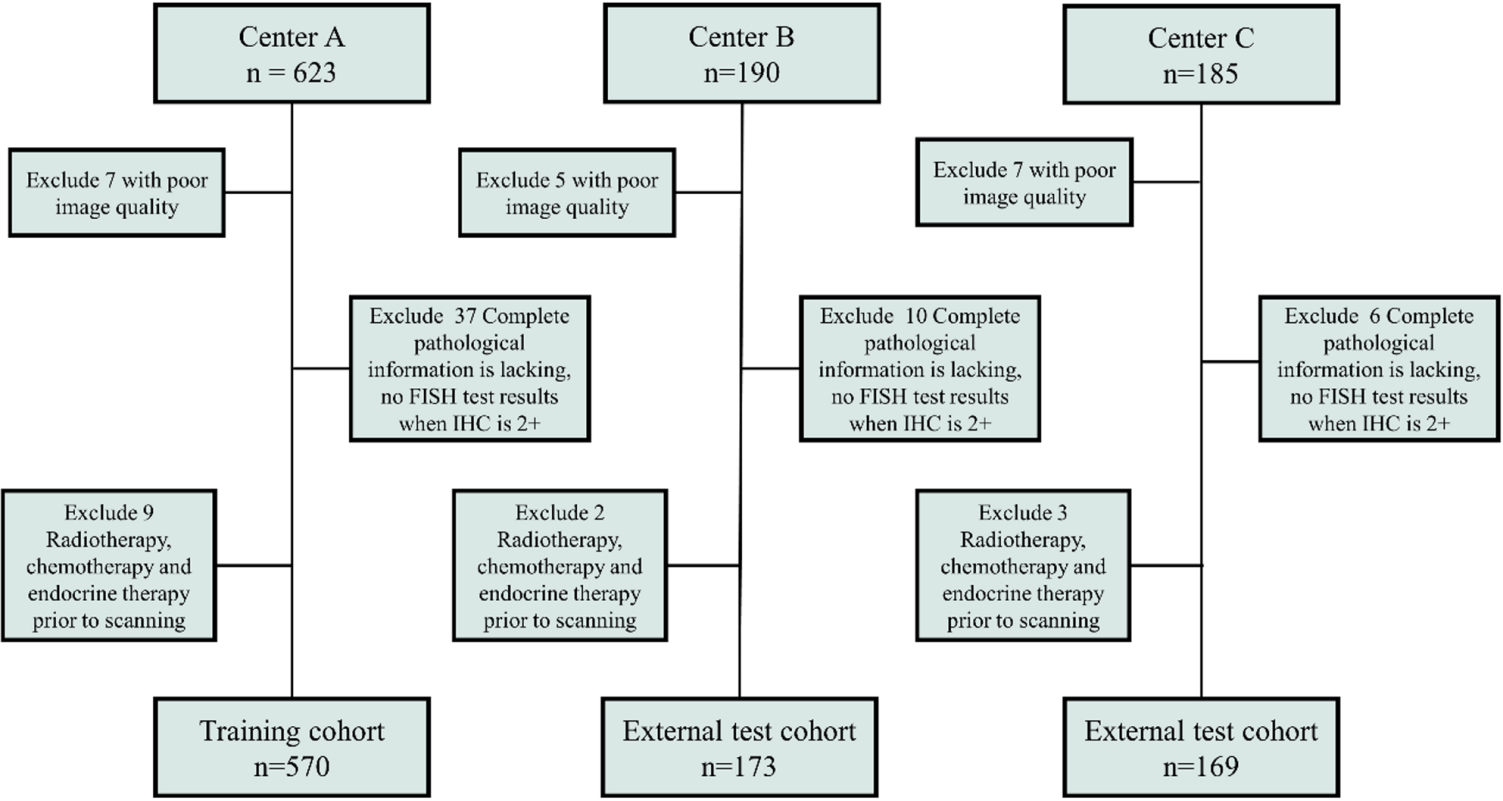
Flowchart shows patient exclusion for each dataset

This study retrospectively collected data from patients who underwent DCE-MRI examinations before treatment at three medical centers, A, B, and C, between April 2018 and March 2024 and were pathologically confirmed to have breast cancer. Inclusion criteria were as follows: (a) Preoperative DCE-MRI examination and (b) breast cancer diagnosed by biopsy after puncture or surgery, with complete HER2 evaluation results. The exclusion criteria were as follows: (a) poor image quality of MRI scans or lack of dynamic contrast enhancement, (b) incomplete pathological information and absence of FISH testing when IHC was 2+, and (c) prior radiotherapy, chemotherapy, and endocrine therapy before scanning (Fig. 1). Pathological data assessment is detailed in the supplementary material, Appendix S1. A total of 912 breast cancer patients who met the inclusion criteria were recruited from the three centers. Center A (*n* = 570) was designated as the training cohort, while Centers B (*n* = 173) and C (*n* = 169) were used as external test cohorts.

### MRI procedure and image preprocessing

Breast MRI examinations were conducted using 1.5T or 3.0T systems (imaging protocols in Appendix S1). Three-phase DCE-MRI was exported from the Picture Archiving Communication System (PACS), specifically in the pre-enhanced, early (2 min after contrast injection), and delayed (approximately 6 min after contrast injection) phases. Image preprocessing involved the application of N4 bias correction [26], resampling, and image normalization (Appendix S1). Two radiologists with five and seven years of work experience delineated the region of interest (ROI) for breast cancer lesions based on the early phases using ITK-SNAP (version 3.8.0; http://www.itksnap.org/pm.s/pm.s.php). The delineated ROIs were then registered in the pre-enhanced and delayed phases.

### Perfusion mapping and tumor subregion clustering

To characterize intratumoral perfusion heterogeneity, we adopted a habitat-based approach that identifies biologically distinct subregions within the tumor. This method assumes that different perfusion patterns—reflecting variations in vascularity, permeability, and microenvironmental activity—correspond to clinically meaningful tumor phenotypes relevant to HER2 biology. Voxel-level perfusion parameters (wash-in, wash-out, and wash-out ratio) from DCE-MRI were combined into a perfusion vector for each voxel. K-means clustering was then applied to group voxels with similar perfusion characteristics into discrete “habitats.” Unlike conventional radiomics, which extracts features from the whole tumor, this approach retains spatially resolved perfusion information and enables subregion-specific radiomics analysis. The resulting habitat features quantify perfusion-driven heterogeneity and provide a more biologically informed representation of tumor behavior than standard single-region radiomics.

**Fig. 2:**
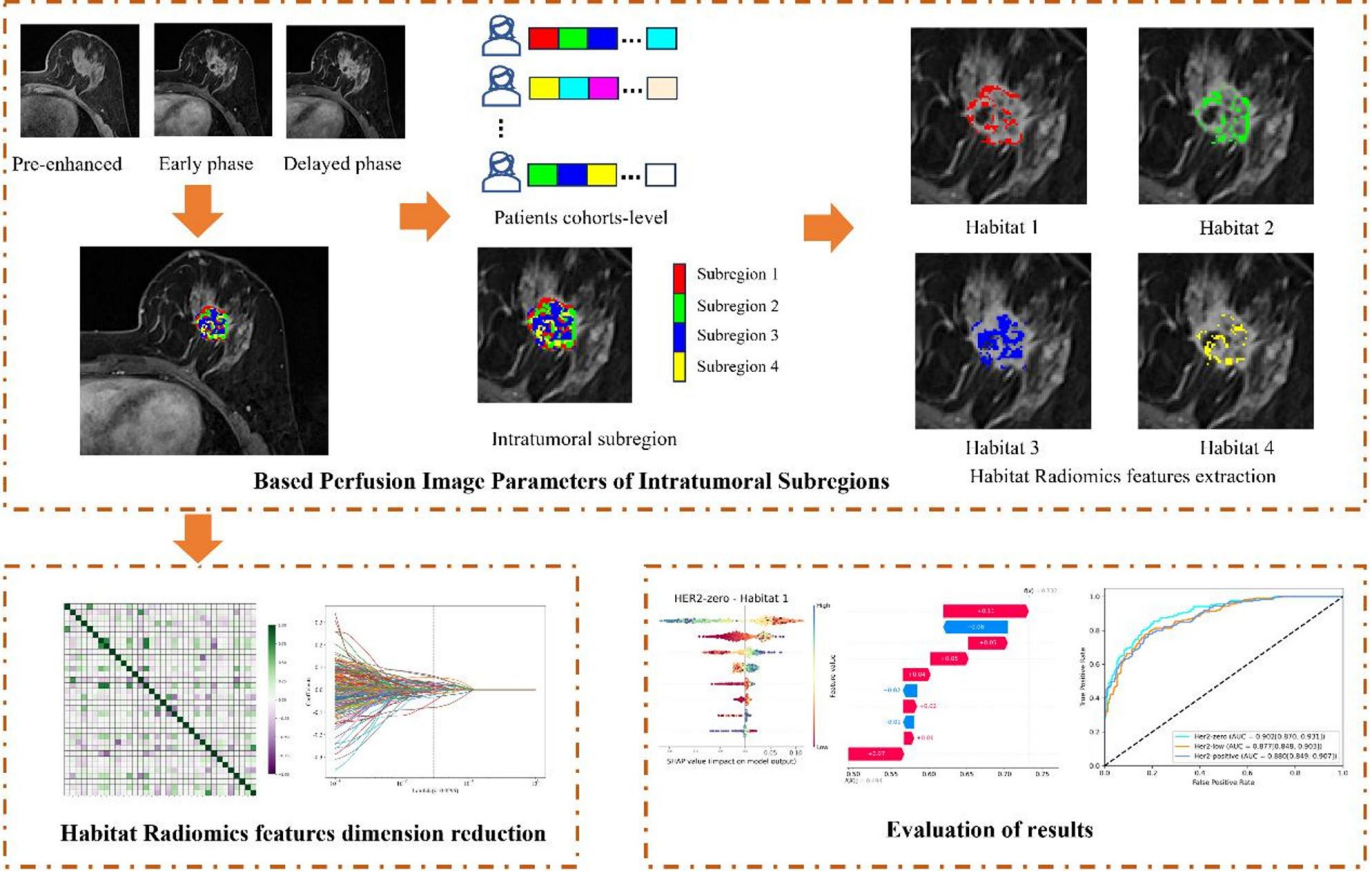
Overview of the framework of the MRI intratumoral perfusion heterogeneity

As shown in Fig. 2, we first constructed three perfusion parametric maps (wash-in, wash-out, and wash-out ratios) using the pre-enhanced, early, and delayed phases of DCE-MRI (Appendix S1). Each perfusion map was computed on a voxel-by-voxel basis to quantify the perfusion characteristics of the tumor tissue at the voxel level. Subsequently, a voxel vector was constructed based on the three perfusion parameters: wash-in, wash-out, and wash-out ratio maps. Each voxel vector in the tumor region contained three perfusion characteristics. We used the k-means clustering algorithm with k values ranging 2–10 to group similar voxel vectors in the tumor images into clusters. The clustering results were evaluated using the Silhouette Coefficient and Davies-Bouldin Index for each value of k (Appendix S1). This task was completed using the OneKey platform (http://www.medai.icu/).

### Feature dimension reduction

We used PyRadiomics (version 3.1; https://pyradiomics.readthedocs.io) to extract 1197 radiomic features from each intratumoral subregion separately [27] (Appendix S1). For partially missing intratumoral subregions, we employed a KNN-based method to impute the missing radiomic features. Each feature was standardized using the z-score normalization method based on the mean and standard deviation of the training cohort. We employed Analysis of Variance (ANOVA), Pearson correlation analysis and the least absolute shrinkage and selection operator (LASSO) method to select radiomic features for the intratumoral subregions.

### Machine learning model development and interpretation

To address the class imbalance issue, we first applied Synthetic Minority Over-sampling Technique (SMOTE) and Random Under Sampler to resample the training data. Subsequently, we used XGBoost, an efficient gradient boosting decision tree algorithm known for its ability to deliver excellent predictive performance. To optimize the XGBoost model’s performance, we employed Grid Search Cross-Validation to tune its hyperparameters. The hyperparameters tuned included the number of trees, the maximum depth of the trees, the learning rate, the sample subset ratio, and the feature subset ratio for each tree. By performing grid search, we identified the best hyperparameter combination, which improved the model’s accuracy and generalization ability.

For model interpretation, we utilized SHAP values for in-depth analysis. SHAP values help us understand the contribution of each feature to the model’s predictions. We used SHAP summary plots to display feature importance, SHAP waterfall plots to explain the prediction results for specific observations. These visualization tools provided detailed insights into the XGBoost model’s prediction mechanisms and enhanced the model’s interpretability. The relevant code has been uploaded to GitHub (https://github.com/Ytdhblkfcdhxla/Breast_Habitate_XGBoost_SHAP).

### Statistical analysis

For continuous data that are normally distributed, we use Analysis of Variance (ANOVA) and present the results as mean$\:\pm\:$standard deviation. For comparisons involving categorical variables, we employ the $\:{x}^{2}$test or Fisher’s exact test and report the findings as frequencies (percentages). In all statistical analyses, a *p*-value less than 0.05 is considered statistically significant. For the ternary classification prediction model, we utilized a one vs. rest strategy. The predictive performance of the HER2 ternary classification model was evaluated using Receiver Operating Characteristic (ROC) curve analysis and confusion matrix. The diagnostic metrics (accuracy, specificity, sensitivity, precision, and F1 score) were obtained by computing a confusion matrix.

## Results

### Patient characteristics

Overall, 912 patients who underwent MRI examinations at the centers were included. The training cohort comprised 570 patients from Centers A (mean age, 49.32 ± 10.87 years). The external testing cohorts consisted of 173 patients from Center B (mean age, 60.47 ± 11.34 years) and 169 patients from Center C (mean age, 49.25 ± 12.28 years). HER2-zero, 264 cases (28.95%); HER2-low, 373 cases (40.90%); HER2-positive, 275 cases (30.15%) in all datasets. Age, history of breast cancer, menstrual status, HR status, molecular subtype, Ki-67 index, and HER2 expression significantly differed among the three datasets (Table 1). The significant differences in clinical data across the three centers provide assurance for the model’s generalizability.

**Table 1:** Characteristics of patients in the center A, B and C

Characteristic	Center A (*n* = 570)	Center B (*n* = 173)	Center C (*n* = 169)	*P* Value
Age (y)	49.32 ± 10.87	60.47 ± 11.34	49.25 ± 12.28	< 0.001
History of breast cancer				< 0.001
Present	41 (7.2)	60 (34.7)	8 (4.7)	
Absent	529 (92.8)	113 (65.3)	161 (95.3)	
Menstrual status				< 0.001
Premenopausal	343 (60.2)	37 (21.4)	73 (43.2)	
Postmenopausal	227 (39.8)	136 (78.6)	96 (56.8)	
Hormone receptor status				0.004
Positive	442 (77.5)	151 (87.3)	124 (73.4)	
Negative	128 (22.5)	22(12.7)	45 (26.6)	
Molecular subtype				0.001
Luminal A	192 (33.7)	49 (28.3)	65 (38.5)	
Luminal B	250 (43.9)	102 (59.0)	59 (34.9)	
HER2 positive	70 (12.3)	11 (6.4)	26 (15.4)	
Triple negative	58 (10.2)	11 (6.4)	19 (11.2)	
Ki-67 index				< 0.001
Low proliferation (< 20%)	175 (30.7)	31 (17.9)	78 (46.2)	
High proliferation (≥ 20%)	395 (69.3)	142 (82.1)	91 (53.8)	
HER2 expression				< 0.001
HER2-zero	118 (20.7)	84 (48.6)	62 (36.7)	
HER2-low	267 (46.8)	48 (27.7)	58 (34.3)	
HER2-positive	185 (32.5)	41 (23.7)	49 (29.0)	

### Performance evaluation of prediction models

This study utilized three perfusion parameters—wash-in, wash-out, and wash-out ratio—to construct voxel-wise perfusion vectors. To characterize perfusion heterogeneity, we applied k-means clustering to these vectors. The optimal number of subregions was determined to be *k = 4* based on the highest Silhouette Coefficient and the lowest Davies–Bouldin Index (Figure S1). A total of 1,197 radiomic features were extracted from each habitat, resulting in 4,788 features across the four subregions. Feature reduction was performed in three steps: ANOVA filtering reduced the candidate set to 1,408 features, Pearson correlation analysis further reduced it to 254 features, and the LASSO method finally selected 34 informative radiomic features (8, 8, 8, and 10 features for Habitats 1–4). The feature selection process is illustrated in Figure S2.

**Fig. 3:**
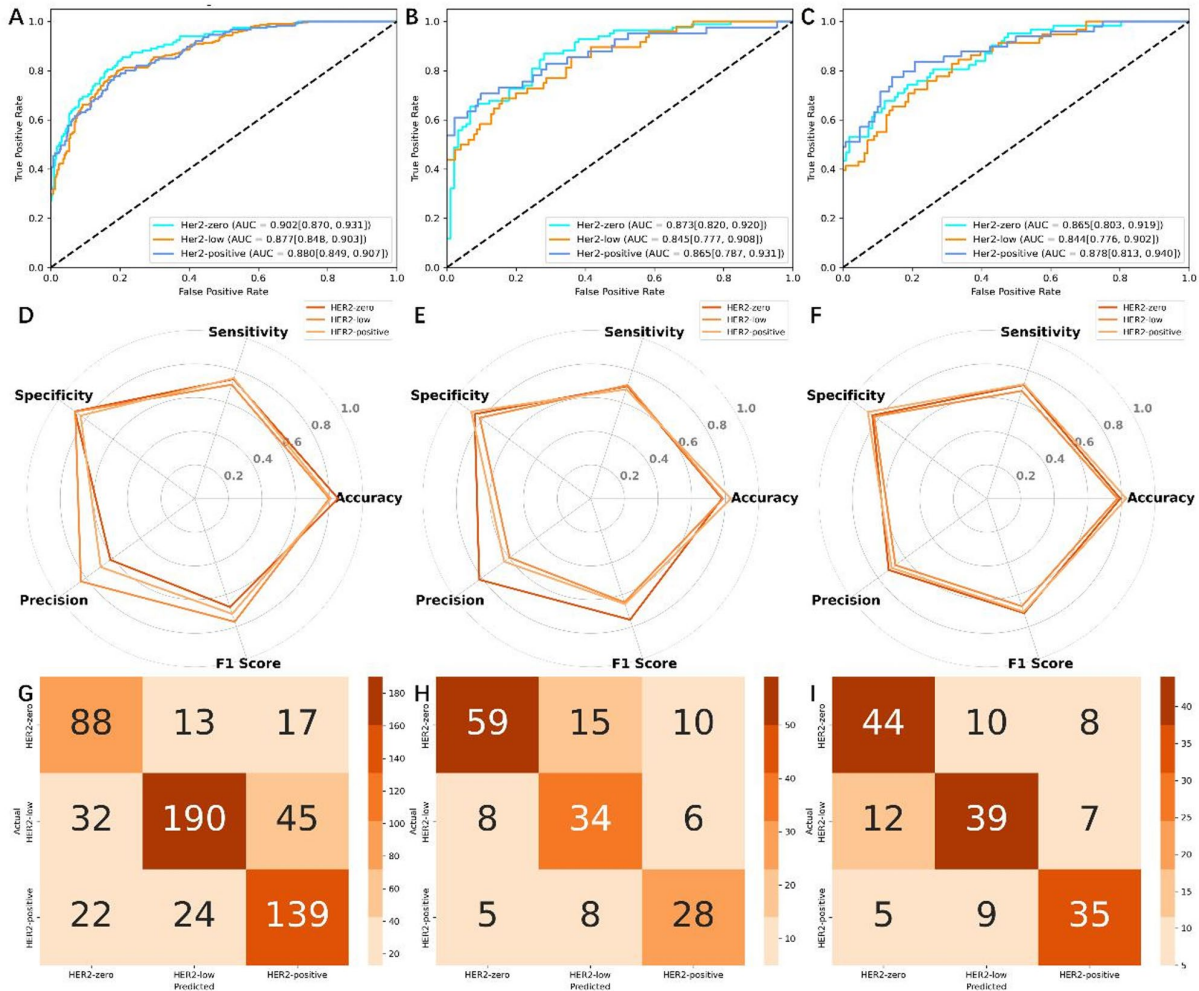
Performance of the habitat model across center **A**, **B**, and **C**. (**A**–**C**) ROC curves. (**D**–**F**) Radar plots of diagnostic metrics. (**G**–**I**) Confusion matrices for the three cohorts

In this study, we employed the XGBoost classifier and optimized the model’s hyperparameters through grid search. The final optimal hyperparameters were: a maximum tree depth of 3, a learning rate of 0.01, 100 trees, and both feature sampling and data sampling ratios set to 60%. In distinguishing the ternary expression status of HER2 in breast cancer, the Habitat model’s performance is assessed by AUC values across multiple centers. At Center A, the AUCs for HER2-zero, HER2-low, and HER2-positive are 0.902 (95% CI: 0.870, 0.931), 0.877 (95% CI: 0.848, 0.903), and 0.880 (95% CI: 0.849, 0.907) respectively. At Center B, the AUCs are reported as 0.873 for HER2-zero (95% CI: 0.820, 0.920), 0.845 for HER2-low (95% CI: 0.777, 0.908), and 0.865 for HER2-positive (95% CI: 0.787, 0.931). At Center C, the AUCs stand at 0.865 for HER2-zero (95% CI: 0.803, 0.919), 0.844 for HER2-low (95% CI: 0.776, 0.902), and 0.878 for HER2-positive (95% CI: 0.813, 0.940). These results are presented in Fig. 3. The radar map in Fig. 3 displays the remaining diagnostic metrics (accuracy, specificity, sensitivity, precision, and F1 score), and Table 2 provides a summary. The confusion matrices for the Habitat models are shown in Fig. 3.

**Table 2:** Discrimination performance comparison of the prediction models

Cohorts	HER2 status	AUC	95% CI	Accuracy	Sensitivity	Specificity	Precision	F1 score
	HER2-zero	0.902	0.870, 0.931	0.853	0.746	0.881	0.620	0.677
Center A	HER2-low	0.877	0.848, 0.903	0.800	0.712	0.878	0.837	0.769
	HER2-positive	0.880	0.849, 0.907	0.811	0.751	0.839	0.692	0.720
	HER2-zero	0.873	0.820, 0.920	0.780	0.702	0.854	0.820	0.756
Center B	HER2-low	0.845	0.777, 0.908	0.786	0.708	0.816	0.596	0.648
	HER2-positive	0.865	0.787, 0.931	0.832	0.683	0.879	0.636	0.659
	HER2-zero	0.865	0.803, 0.919	0.793	0.710	0.841	0.721	0.715
Center C	HER2-low	0.844	0.776, 0.902	0.775	0.672	0.829	0.672	0.672
	HER2-positive	0.878	0.813, 0.940	0.828	0.714	0.875	0.700	0.707

### Explanation and visualization of habitat model

**Fig. 4:**
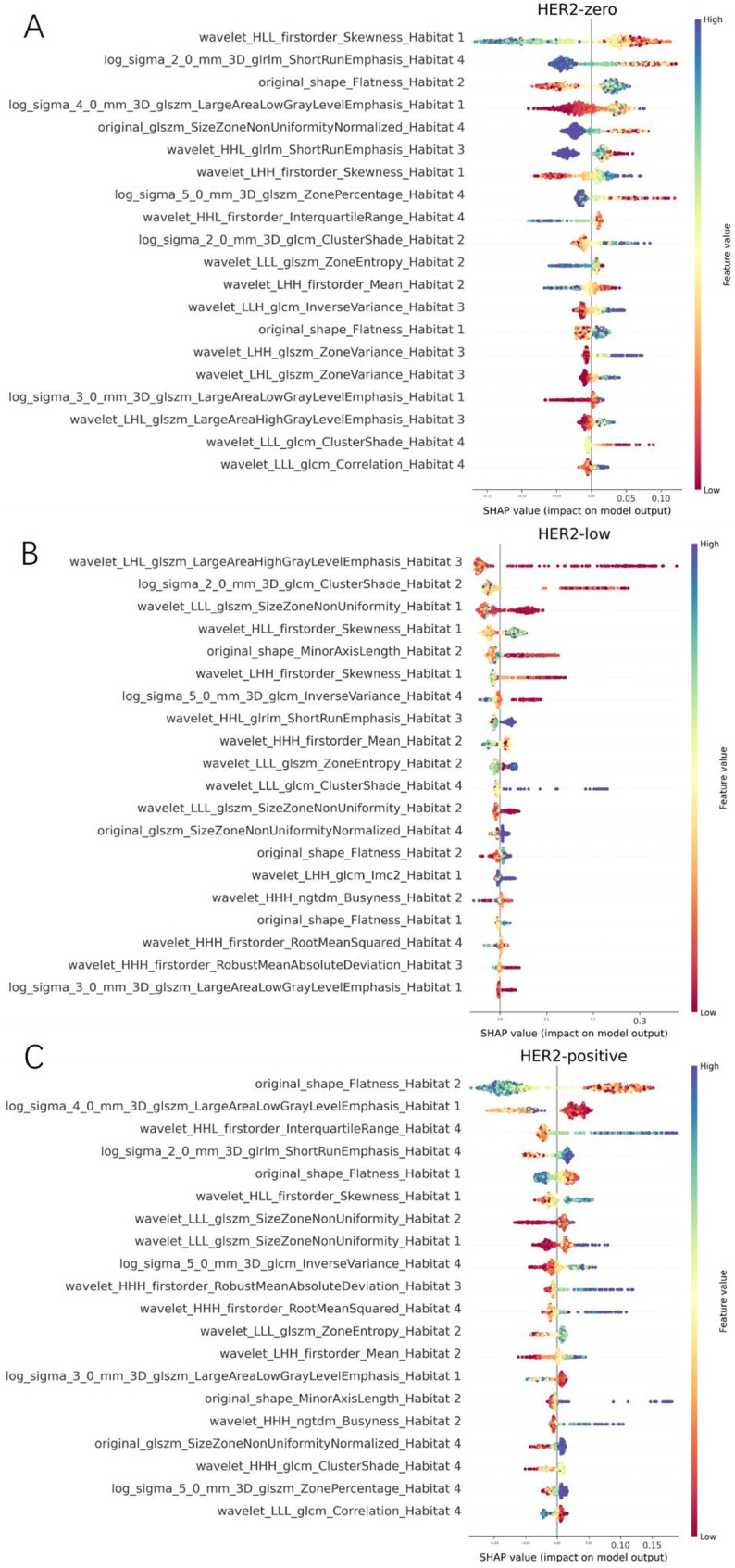
SHAP summary plots showing the most influential radiomics features for predicting HER2-zero, -low, and -positive status. Only the top 20 features are displayed

This study employs the SHAP method to interpret the model’s predictions, offering both global and local explanations. The global explanation is illustrated through SHAP summary plots, such as those shown in Fig. 4, which highlight feature importance and influence. Features are ranked by their average SHAP values and are color-coded to represent the size of the feature values (ranging from low [red] to high [blue]), showcasing the overall impact of each feature on the model’s predictions. For predicting HER2 null, low, and high expression statuses in breast cancer, the most important features are wavelet_HLL_firstorder_Skewness_Habitat1, wavelet_LHL_glszm_LargeAreaHighGrayLevelEmphasis_Habitat3, and original_shape_Flatness_Habitat2, respectively. The contribution direction and magnitude of these features vary considerably across different samples. Specifically, as illustrated in Fig. 4A, a decrease in wavelet_HLL_firstorder_Skewness_Habitat1 enhances its positive contribution to predicting HER2 null expression. The figure displays only the top 20 most significant features, with additional features shown in Supplementary Material Figure S3.

Beyond identifying statistically important radiomic features, the Habitat model also revealed clinically meaningful patterns associated with perfusion heterogeneity. The most influential features across the four subregions were primarily derived from wavelet- and texture-based descriptors, reflecting structural, vascular, and microenvironmental variations within tumors. For example, wavelet_HLL_firstorder_Skewness from Habitat 1, a subregion characterized by rapidly enhancing voxels, may reflect asymmetric perfusion intensity associated with necrosis or heterogeneous vascular supply, which is more common in HER2-zero tumors. Similarly, texture measures such as wavelet_LHL_glszm_LargeAreaHighGrayLevelEmphasis from Habitat 3 capture regional signal homogeneity and may correspond to densely vascularized tissue that is often seen in HER2-low disease. Original_shape_Flatness features derived from Habitat 2, such as Flatness, may indicate compressive effects or directional tumor growth patterns associated with HER2-positive biology.

**Fig. 5:**
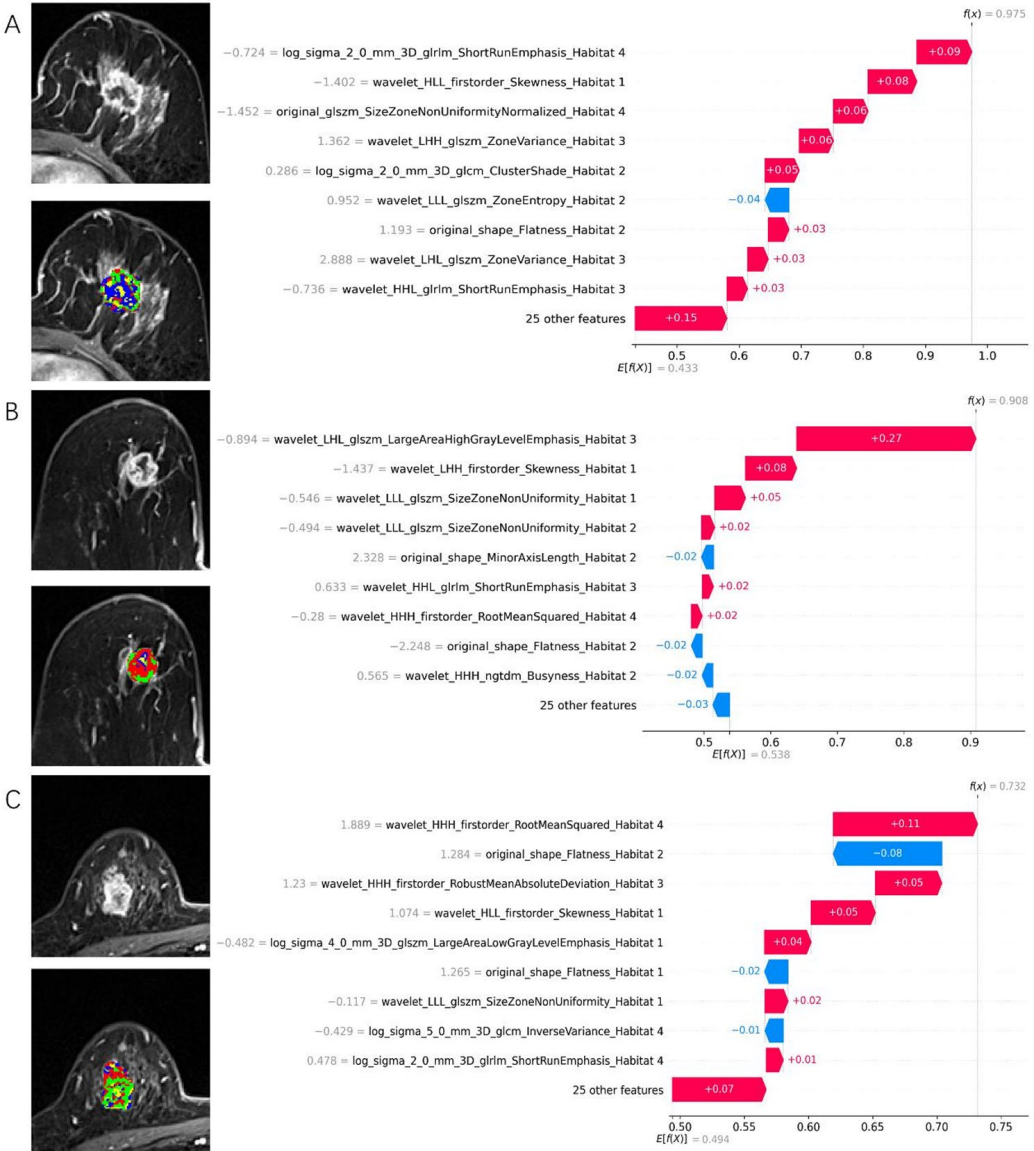
SHAP waterfall plots illustrating local explanations for three representative patients with HER2-zero (**A**), HER2-low (**B**), and HER2-positive (**C**) status

Additionally, local explanations are provided by analyzing SHAP values for individual samples to understand the reasons behind specific predictions, enhancing the model’s interpretability. Figure 5 show SHAP waterfall plots for three breast cancer patients, representing HER2-zero, HER2-low, and HER2-positive statuses. For example, Fig. 5A demonstrates that the feature combination for this observation significantly increased the model’s probability of predicting HER2-zero (from 0.433 to 0.975). Features such as log_sigma_2_0_mm_3D_glrlm_ShortRunEmphasis_ Habitat4 (with a SHAP value of 0.09 and colored red) strongly contribute to predicting HER2-zero, while wavelet_LLL_glszm_ZoneEntropy_ Habitat2 (with a SHAP value of 0.04 and colored blue) contributes to predicting non-HER2-zero states. Similarly, Fig. 5B shows a significant increase in HER2-low prediction probability (from 0.538 to 0.908) due to the feature combination, while Fig. 5C indicates a notable rise in HER2-positive prediction probability (from 0.494 to 0.732). These plots highlight how specific feature combinations can substantially influence prediction probabilities for different HER2 statuses.

## Discussion

This study demonstrated that perfusion-based habitats extracted from DCE-MRI can predict HER2-zero, -low, and -positive expression with high performance across multicenter datasets.

Compared with existing MRI-based radiomics and deep-learning studies on HER2 characterization, our approach provides several methodological advantages. Prior work has typically analyzed whole-tumor radiomic features or relied on binary or pairwise classification frameworks, which may overlook spatially distinct perfusion phenotypes and dilute clinically relevant signals [24, 28]. Deep-learning–based models have demonstrated moderate performance but similarly did not explicitly account for voxel-level heterogeneity or differences across HER2 subgroups [25]. In contrast, our habitat-based framework leverages voxel-level perfusion clustering to isolate biologically meaningful subregions before feature extraction, enabling the model to capture perfusion-driven heterogeneity that aligns with known HER2-related vascular and microenvironmental patterns. Furthermore, integrating SHAP provides transparent, biologically interpretable insights that many prior radiomics and deep-learning models lack.

HER2 overexpression increases cell proliferation, survival, motility, and invasiveness and enhances the production of vascular endothelial growth factors to promote angiogenesis [29]. Tumor perfusion heterogeneity can indirectly reflect the expression status of HER2 in breast cancer [30]. Therefore, we hypothesized that intratumoral spatial heterogeneity may be reflected in the tumor’s blood perfusion on DCE-MRI, allowing for analysis through data-driven clustering based on this feature. This is also the basis for selecting wash-in, wash-out, and wash-out ratio parameters from DCE-MRI to form voxel vectors, enabling tumor segmentation into multiple regions.

As molecular pathological tests directly targeting HER2 protein or gene, IHC/FISH have mature detection efficacy for HER2 overexpression (IHC 3+/FISH+) and serve as the core basis for traditional anti-HER2 treatment decisions, but they have inherent limitations: relying on invasive tissue sampling (≈ 20%-30% of advanced patients cannot undergo it due to biopsy contraindications), being prone to sampling bias from intratumoral heterogeneity (≈ 15%-20% of HER2-positive tumors may be misjudged), and having ambiguous thresholds for distinguishing HER2-zero from HER2-low (up to 40% inconsistency between primary and metastatic lesions) [31]. This study’s model addresses these shortcomings: based on whole-tumor analysis of DCE-MRI perfusion subregions, it enables non-invasive assessment without tissue samples. In external validation cohorts, its predictive specificities for HER2-zero and HER2-positive reached 84.1%-85.4% and 87.5%-87.9% respectively. Moreover, by capturing perfusion heterogeneity, it achieves direct ternary classification of HER2-zero, -low, -positive (AUC = 0.845 and 0.844 for HER2-low in external validation B and C), breaking IHC/FISH’s bottleneck in distinguishing low HER2 expression.

This study, based on the XGBoost model, developed the Habitat model, which demonstrated strong performance. The XGBoost model is widely used in developing clinical and imaging models due to its robust capabilities [32, 33]. understanding the model’s mechanism and interpretability is crucial. The SHAP method offers explanations and visualizations for the XGBoost model using SHAP summary plots and waterfall plots, presenting them in a simple and comprehensible manner. This helps clinicians better understand and apply the model. In this study, SHAP effectively illustrates the impact of features on different HER2 expression statuses, with the range and color of the points indicating the affected features for each status. For breast cancer HER2 expression—zero, low, and positive—the most important predictive feature originates from different tumor subregions: Habitat 1, Habitat 3, and Habitat 2, respectively. Furthermore, radiomic features within the same subregion contribute differently to distinguishing HER2 expression levels. For instance, the radiomic feature wavelet_HLL_firstorder_Skewness_Habitat 1, derived from Habitat 1, has the highest average absolute SHAP value among features in this subregion when predicting HER2-zero expression. However, its contribution ranks second for HER2-low expression and third for HER2-positive expression. These findings further underscore the pivotal role of tumor spatial heterogeneity in predicting HER2 expression status in breast cancer and highlight the distinct contributions of different tumor subregions to HER2 classification. For evaluating the impact of features on individual breast cancer patients, this study provides local explanations using waterfall plots. Compared to traditional nomogram methods, SHAP force plots are more efficient and user-friendly [34]. In the model provided, if the SHAP value exceeds the baseline, the patient can be classified into the corresponding category. Furthermore, by examining the color and length of the arrows, one can understand the influence of features on the patient’s assessment, with the length of the arrow representing the contribution of specific features to the evaluation [35].

This study had some limitations owing to its retrospective nature. Although two external validations were collected to enhance reliability, further prospective analyses are needed. Manual delineation of tumors by different readers may affect radiomic features’ stability.

This study developed a habitat model based on DCE-MRI perfusion heterogeneity that predicted the ternary expression status of breast cancer HER2. It can potentially become a noninvasive tool for identifying HER2 expression status. Additionally, the SHAP method can interpret the results generated by the model, providing an interpretable framework for clinical applications. It offers valuable explanations for each patient’s prediction outcome, helping to understand the sources and rationale behind the predictions.

## Supplementary Information

Below is the link to the electronic supplementary material.

Supplementary Material 1

## Data Availability

The datasets used and/or analysed during the current study available from the corresponding author on reasonable request.

## Code availability

The underlying code for this study is available in GitHub and can be accessed via this link https://github.com/Ytdhblkfcdhxla/Breast_Habitate_XGBoost_SHAP.

## Abbreviations

HER-2: Human epidermal growth factor receptor 2
SHAP: SHapley Additive exPlanations
XGBoost: Extreme Gradient Boosting
IHC: Immunohistochemistry
FISH: Fluorescence in situ hybridization
ADC: Antibody-drug conjugate
MRI: Magnetic resonance imaging
DCE: Dynamic contrast-enhanced
ER: Estrogen receptor
PR: Progesterone receptor
HR: Hormone receptor
PACS: Picture Archiving Communication System
ROI: Region of interest
LASSO: Least absolute shrinkage and selection operator
KNN: K-Nearest Neighbors
LR: Logistic Regression
ROC: Receiver Operating Characteristic
AUC: Receiver Operating Characteristic curve
