# Supplementary material for: A SHAP-interpretable XGBoost model: MRI-based intratumoral perfusion heterogeneity predicts HER2-zero, -low, and -positive ternary expression status in breast cancer
Source: Cancer Imaging. 2026 Feb 2;26:35. doi: 10.1186/s40644-026-01000-4 (PMC12954891; doi:10.1186/s40644-026-01000-4)
Supplement: Supplementary file 1 — Supplementary Material 1 [file 40644_2026_1000_MOESM1_ESM.docx]

**Appendix S1**

**Imaging Protocols**

For Center A, all MRI scans were obtained using Siemens Avanto 1.5T and Skyra 3.0T MRI scanners, utilizing a dedicated breast coil. Pre-contrast fat-saturated T1-weighted imaging (T1WI) was collected before the injection of the contrast agent. Dynamic contrast-enhanced T1-weighted (T1+C) images included five post-contrast scans, collected at 60-second intervals after the intravenous injection of gadolinium contrast agent. A gadolinium-based agent was injected using a power injector at a rate of 3 mL/s, with a dose of 0.2 mmol/kg of body weight, followed by a 20 mL saline flush administered with a high-pressure injector.

For Center B, all MRI scans were obtained using Siemens Avanto 1.5T MRI scanners, utilizing a dedicated breast coil. Pre-contrast fat-saturated T1-weighted imaging (T1WI) was collected before the injection of the contrast agent. Dynamic contrast-enhanced T1-weighted (T1+C) images included five post-contrast scans, collected at 60-second intervals after the intravenous injection of gadolinium contrast agent. A gadolinium-based agent was injected using a power injector at a rate of 3 mL/s, with a dose of 0.2 mmol/kg of body weight, followed by a 30 mL saline flush administered with a high-pressure injector.

For Center C, all MRI scans were obtained using 1.5T Philips (Achieva) or 3.0T Siemens (Prisma and Skyra) MRI scanners, utilizing a dedicated breast coil. Pre-contrast fat-saturated T1-weighted imaging (T1WI) was collected before the injection of the contrast agent. Dynamic contrast-enhanced T1-weighted (T1+C) images included five post-contrast scans, collected at 60-second intervals after the intravenous injection of gadolinium contrast agent. A gadolinium-based agent was injected using a power injector at a rate of 2 mL/s, with a dose of 0.2 mmol/kg of body weight, followed by a 20 mL saline flush administered with a high-pressure injector.

**Pathological data assessment**

HER2 status was determined using IHC and FISH according to the clinical practice guidelines for HER2 testing in breast cancer issued by the American Society of Clinical Oncology/College of American Pathologists. IHC results of 2+ were considered ambiguous and were further tested by FISH. The HER2 results were categorized as HER2 zero expression (IHC score 0), HER2 low expression (IHC score 1+ or 2+ without FISH amplification), and HER2 overexpression (IHC score 3+ or 2+ with FISH amplification). Additionally, Estrogen receptor (ER) and Progesterone receptor (PR) positivity was an IHC expression level of ≥1%. Hormone receptor (HR)-positive status was called ER or PR positivity. The cutoff value for the Ki67 index is 20%, delineating low expression (<20%) from high expression (≥20%). Breast cancer is classified as luminal A, luminal B, HER2-positive, and triple-negative based on ER, PR, HER-2 status, and the tumor proliferation index Ki-67.

**Imaging Data Preprocessing**

This study includes MRI scan data from three different medical centers. Differences in spatial resolution, imaging protocols, image quality, and artifacts across centers may affect the model's generalizability. Therefore, we first applied N4 bias field correction to correct low-frequency bias fields in the MRI images, improving image quality. Next, we used nearest neighbor resampling to standardize the images to a resolution of 1×1×1 mm³. Finally, to enhance contrast, we normalized the image data to the range [0, 255]. Specifically, this normalization is achieved indirectly by calculating the mean and standard deviation of the image gradients, meaning that the normalization process is applied to the gradient images rather than the original image data.

**Radiomic Feature Extraction**

This study employs PyRadiomics, a Python-based toolkit for radiomics feature extraction, version 3.0, to extract features from MRI images. Custom feature extraction is facilitated through a configuration file that is divided into two key sections: imageType, and featureClass.

Under the imageType section, the imagetype defines data augmentation operations. Original signifies that no alterations are made to the original image, and features are extracted as is. LoG represents Laplacian of Gaussian transformation, which involves two steps: Gaussian denoising followed by Laplacian transformation, with the sigma parameter dictating the extent of blurring during the denoising step. Wavelet stands for wavelet transformation, where the choice of wavelet function is specified.

The featureClass section specifies the categories of features to be extracted. This includes shape-based features (shape), encompassing 3D and 2D shape features with 14 attributes; first-order statistics (firstorder) totaling 18 features; Gray Level Dependence Matrix (GLDM) with 14 features; Gray Level Co-Occurrence Matrix (GLCM) with 22 features; Gray Level Size Zone Matrix (GLSZM) with 16 features; Neighboring Gray Tone Difference Matrix (NGTDM) with 5 features; and Gray Level Run Length Matrix (GLRLM) with 16 features. All feature classes, except for shape, can be computed on either the original image or derivative images obtained by applying filters-in this case, wavelet and Gaussian filters are used. Shape descriptors are derived independently of gray values from label masks.

**Generation of Perfusion Maps**

The wash-in map is generated by subtracting the pre-enhanced image from the early phases image. The wash-out map is generated by subtracting the delayed phases image from the early phases image. The wash-out ratio is calculated as the ratio of the difference in signal intensity between the early phases and delayed phases to the signal intensity of the early phases. We completed this task using the components of the Onekey platform (http://www.medai.icu/). The calculation formulas are as follows:

$$Wash in=I_{\mathrm{Early}} -I_{\mathrm{Pre}}$$

$$Wash out =I_{\mathrm{Early}} -I_{\mathrm{Delayed}}$$

$$Wash out Ratio= \frac{I_{\mathrm{Early}} -I_{\mathrm{Delayed}}}{I_{\mathrm{Early}}}$$

**Optimal Tumor Subregion**

This study assessed voxel-level clustering results using two methods, the Silhouette Coefficient and the Davies-Bouldin Index, to determine the optimal number of sub-regions. Both metrics are widely used for evaluating clustering quality. The Silhouette Coefficient ranges from -1 to 1, where higher values indicate better clustering quality. A value close to 1 signifies strong intra-cluster cohesion and clear inter-cluster separation, while a value near 0 suggests that data points are at the boundary of clusters, and a value close to -1 indicates possible misassignment to incorrect clusters. Conversely, the Davies-Bouldin Index should be as low as possible, with lower values reflecting superior clustering quality through higher inter-cluster separation and intra-cluster cohesion. Ideally, the Silhouette Coefficient should approach 1, and the Davies-Bouldin Index should be as close to 0 as possible (Figure S1).

**Feature Dimension Reduction**

First, radiomics features from the subregions were filtered using Analysis of Variance (ANOVA), resulting in 1408 features. Subsequently, Pearson correlation analysis was applied for further selection, leaving 254 features. Finally, the Least Absolute Shrinkage and Selection Operator (LASSO) method was used, resulting in 34 remaining radiomics features. Among these, Habitat 1, 2, 3, and 4 each comprised 8, 8, 8, and 10 features, respectively (Figure S2).

**Figures S1**


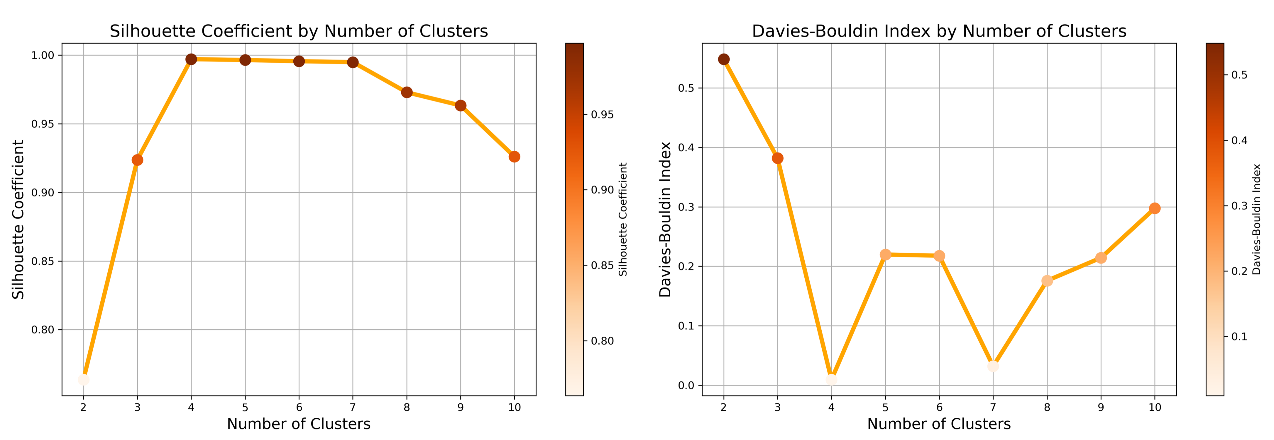


The k-means clustering algorithm is employed with k values ranging from 2 to 10 to group similar voxel vectors in tumor images into clusters. When k was set to four, the Silhouette Coefficient was closest to 1 and the Davies-Bouldin Index was closest to 0, indicating that dividing the images into four sub-regions was optimal.

**Figures S2**


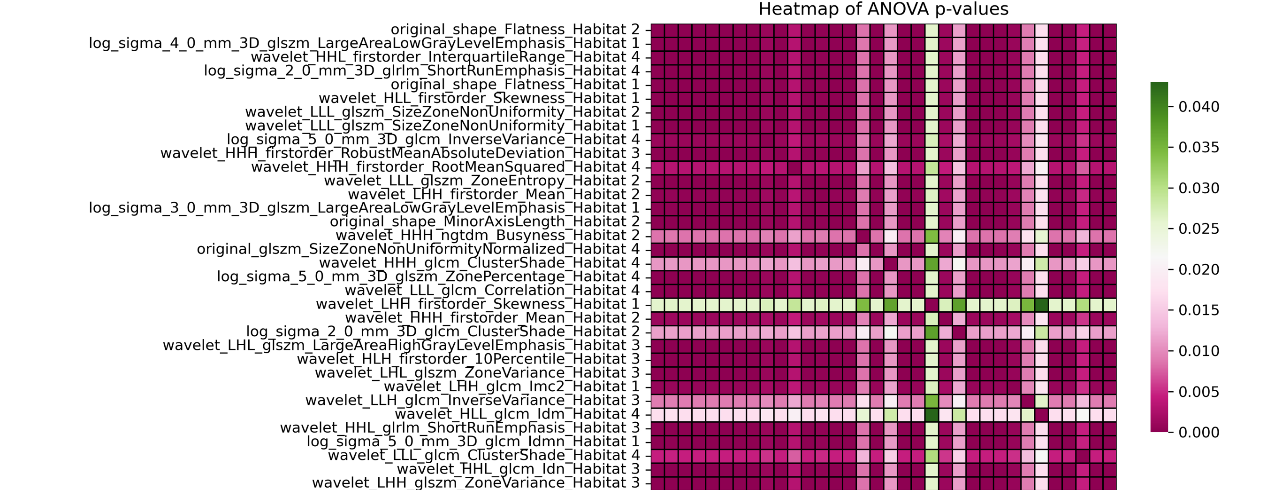


The 34 most valuable radiomics features are highlighted, with heatmaps in the bottom right and top left corners representing the ANOVA p-values of these radiomics features, respectively.


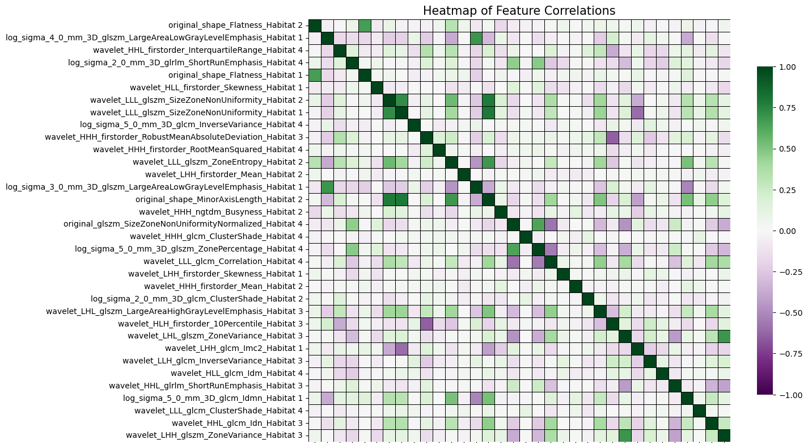


The best radiomics features were selected by Pearson correlation analysis.


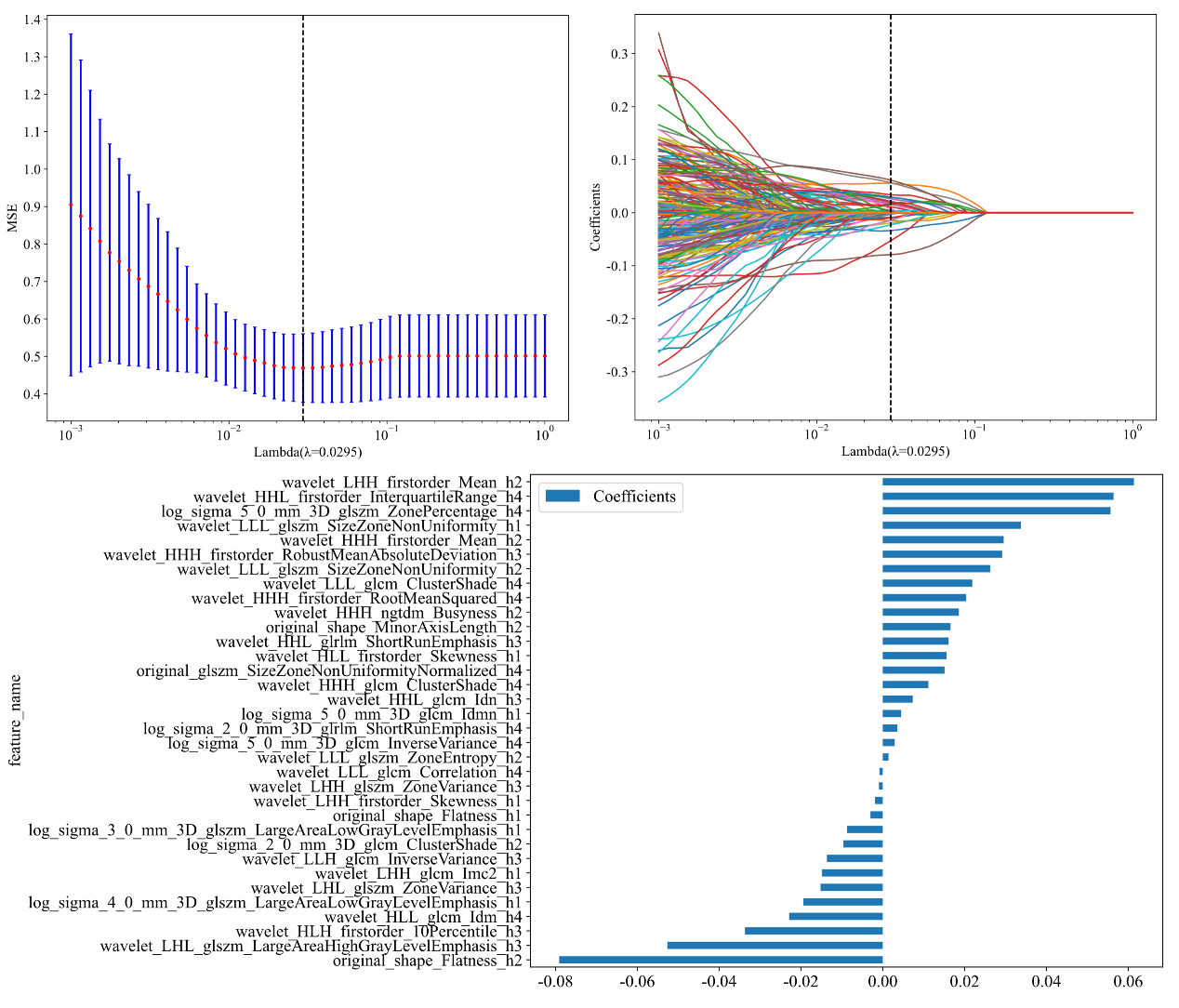


The best radiomics features were selected by LASOO regression and the weight maps for each feature.

**Figures S3**


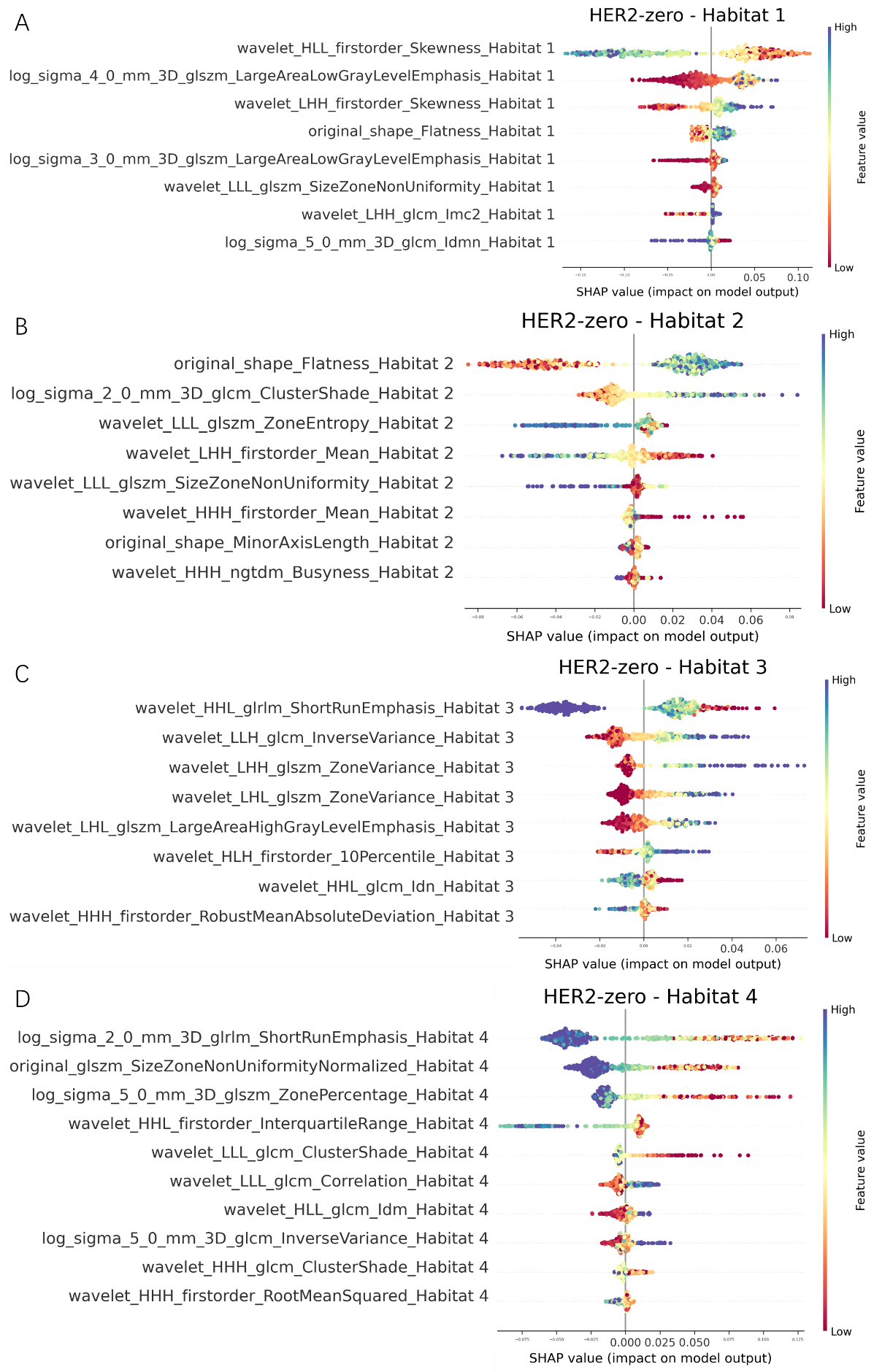


The SHAP summary feature plot for the Habitat model predicting HER2-zero status shows the highlight features for each sub-region. A, B, C, and D display the most important features for Habitat 1, Habitat 2, Habitat 3, and Habitat 4, respectively.


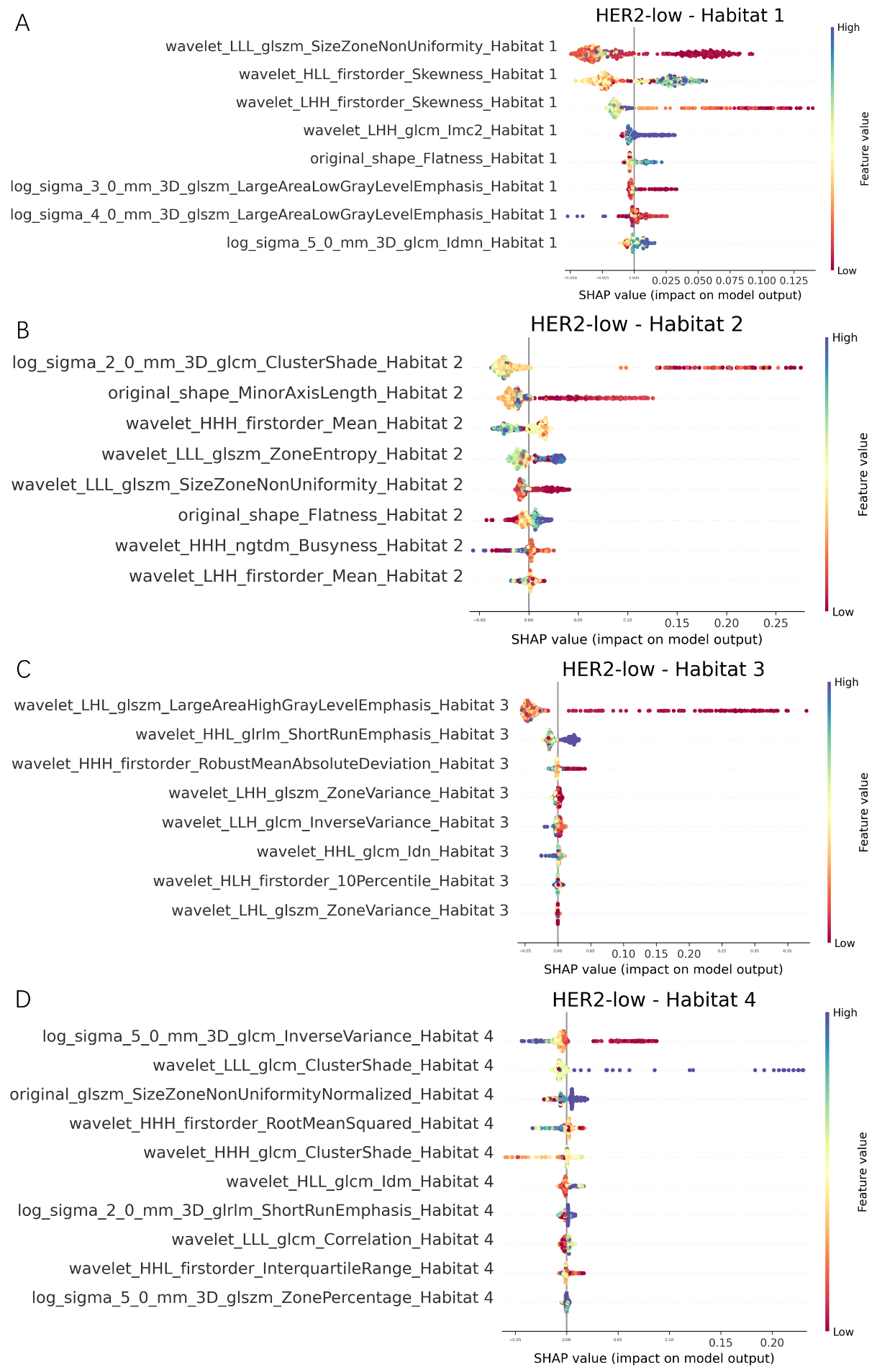


The SHAP summary feature plot for the Habitat model predicting HER2-low status shows the highlight features for each sub-region. A, B, C, and D display the most important features for Habitat 1, Habitat 2, Habitat 3, and Habitat 4, respectively.


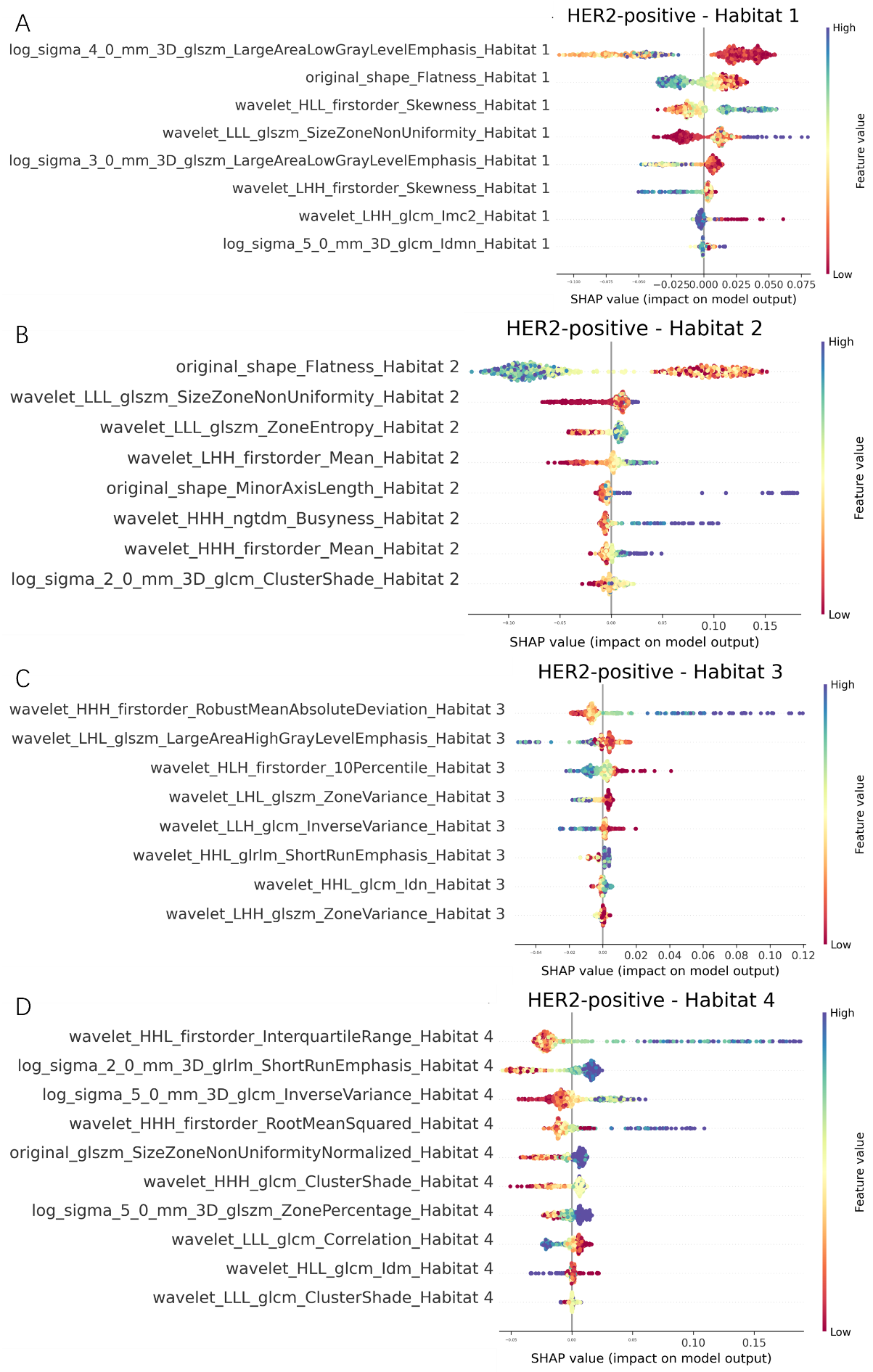


The SHAP summary feature plot for the Habitat model predicting HER2-positive status shows the highlight features for each sub-region. A, B, C, and D display the most important features for Habitat 1, Habitat 2, Habitat 3, and Habitat 4, respectively.
